# Landscape of fear or landscape of food? Moose hunting triggers an antipredator response in brown bears

**DOI:** 10.1002/eap.2840

**Published:** 2023-03-26

**Authors:** Ludovick Brown, Andreas Zedrosser, Jon M. Arnemo, Boris Fuchs, Jonas Kindberg, Fanie Pelletier

**Affiliations:** ^1^ Département de biologie Université de Sherbrooke Sherbrooke Canada; ^2^ Department of Natural Sciences and Environmental Health University of South‐Eastern Norway Bø in Telemark Norway; ^3^ Institute for Wildlife Biology and Game Management University for Natural Resources and Life Sciences Vienna Austria; ^4^ Department of Forestry and Wildlife Management Inland Norway University of Applied Sciences Koppang Norway; ^5^ Department of Wildlife, Fish and Environmental Studies Swedish University of Agricultural Sciences Umeå Sweden; ^6^ Norwegian Institute for Nature Research Trondheim Norway

**Keywords:** behavior, disturbance, human–wildlife interactions, risk perception, scavenger, step‐selection function, *Ursus arctos*

## Abstract

Hunters can affect the behavior of wildlife by inducing a landscape of fear, selecting individuals with specific traits, or altering resource availability across the landscape. Most research investigating the influence of hunting on wildlife resource selection has focused on target species and less attention has been devoted to nontarget species, such as scavengers that can be both attracted or repelled by hunting activities. We used resource selection functions to identify areas where hunters were most likely to kill moose (*Alces alces*) in south‐central Sweden during the fall. Then, we used step‐selection functions to determine whether female brown bears (*Ursus arctos*) selected or avoided these areas and specific resources during the moose hunting season. We found that, during both day and nighttime, female brown bears avoided areas where hunters were more likely to kill moose. We found evidence that resource selection by brown bears varied substantially during the fall and that some behavioral changes were consistent with disturbance associated with moose hunters. Brown bears were more likely to select concealed locations in young (i.e., regenerating) and coniferous forests and areas further away from roads during the moose hunting season. Our results suggest that brown bears react to both spatial and temporal variations in apparent risk during the fall: moose hunters create a landscape of fear and trigger an antipredator response in a large carnivore even if bears are not specifically targeted during the moose hunting season. Such antipredator responses might lead to indirect habitat loss and lower foraging efficiency and the resulting consequences should be considered when planning hunting seasons.

## INTRODUCTION

Animals select resources based on a trade‐off between fear and fitness (Lima & Bednekoff, 1999). Predators, including humans, commonly create a landscape of fear, and potential prey adapt their behavior to modulate their exposure to the perceived risks (Brown et al., 1999; Lima & Bednekoff, 1999; Lima & Dill, 1990). For example, elk (*Cervus elaphus*) in Alberta, Canada, tend to increase their use of concealed areas and move further away from roads during the hunting season compared with the nonhunting season (Paton et al., 2017). Studies on multiple taxa, including fish and mammals, have also reported that artificial removal may apply a demographic filter to a population by selectively harvesting individuals with specific behavioral traits (Leclerc et al., 2017). Ciuti et al. (2012) showed that hunters from southwest Alberta, Canada, harvested elk that were bolder, used open areas more often and had higher movement rates. Hunting can also affect the behavior of nontarget species, for example, opportunistic scavengers, by altering resource availability across the landscape and providing essential resources (Cozzi et al., 2015; Gomo et al., 2017; Lafferty et al., 2016; Wilson et al., 2017). In this study, we determined the response of an opportunistic omnivore to landscape variations in scavenging opportunities and perceived mortality risk from ungulate harvest.

Hunters are not randomly distributed on the landscape and generally use areas based on accessibility (e.g., road access and topography [Diefenbach et al., 2005]) and visibility (e.g., open areas [Lebel et al., 2012]). For example, Lebel et al. (2012) found that the probability of hunters killing a white‐tailed deer (*Odocoileus virginianus*) on Anticosti Island (Québec, Canada) increased with visibility and decreased with increasing distance from roads. Also, harvest sites of brown bear (*Ursus arctos*) and moose (*Alces alces*) are found in close association with roads in both Canada and Sweden (Boer, 1990; Steyaert et al., 2016). Therefore, disturbances or attractants resulting from hunting activities, such as ungulate slaughter remains (i.e., gut piles, bone and hide dumps, or carcasses of wounded animals that were not found) are also concentrated around areas preferentially used by hunters. Scavengers should be attracted to those areas during the ungulate hunting season.

Brown bears are large opportunistic omnivores that are hunted in Sweden. Previous studies have shown that individuals adjust their behavior in response to the bear hunting season by changing their activity pattern and by avoiding areas with high levels of human activities (Hertel, Zedrosser et al., 2016; Ordiz et al., 2011). The bear hunting season in Sweden generally starts ~2 weeks prior to the moose hunting season in the fall; however, we do not know if and how bears react to moose hunting activities. There are two alternative hypotheses predicting behavioral changes in bears in response to moose hunting. First, moose hunters could be perceived as a threat, due to the high amount of human activity on the landscape and therefore brown bears could avoid the areas used by moose hunters due to the perceived risk. Alternatively, moose hunters and their activities could attract bears through the provision of slaughter remains. Swedish hunters harvest ~84,000 moose each year during the fall, which represents ~26% of the population at the national level (Kalén et al., 2022). Thus, large amounts of slaughter remains are available to scavengers during the hunting season. Bears commonly scavenge on slaughter remains discarded by hunters (Elfström et al., 2014; Friebe et al., 2001; Lafferty et al., 2016), and dietary analyses in Scandinavian brown bears showed that ~14% of scats collected during the fall contained vertebrate materials (Stenset et al., 2016). Scandinavian brown bears rely mostly on berries (e.g., *Vaccinium* spp., *Empetrum* spp.) during hyperphagia in the fall (Stenset et al., 2016), but consume meat when available (Dahle et al., 1998; Elfström et al., 2014; Persson et al., 2001). Therefore, we can expect brown bears to scavenge on slaughter remains discarded by moose hunters during the fall.

The goal of this study was to determine whether hunting activities affect the behavior of a nontarget species. We used data from Sweden to evaluate if and how brown bears react to moose hunting because they could perceive this activity as both a threat and a food source. First, we estimated resource selection by moose hunters and determined the relative probability of moose kills across our study area. Second, we evaluated the impact of moose hunting on brown bear habitat selection. We hypothesized that bear habitat selection should vary with hunting activities shifting from bear to moose hunting because the trade‐off between increased mortality risk corresponds with a pulse in resource availability and accessibility. In addition, human activities strongly influence the behavior of bears (Hertel, Zedrosser, et al., 2016; Lamb et al., 2020; Stillfried et al., 2015). We predicted that during the moose hunting season, bears should reduce the use of areas with a higher probability of moose kills during the day (i.e., the legal hunting hours for moose in Sweden); and that they should increase the use of those areas at night to access slaughter remains when humans are inactive on the landscape and because mammals in a general shift to nocturnality to avoid interactions with humans (Gaynor et al., 2018).

## MATERIALS AND METHODS

### Study area

The study area was in Dalarna, Gävleborg and the southern part of Jämtland counties in south‐central Sweden (~61° N, 15° E). The landscape is covered with a highly managed boreal forest with low human density (4–7 inhabitants/km^2^) and a dense network of forest roads (0.7 km/km^2^) (Martin et al., 2010; Ordiz et al., 2013). The main habitat types are coniferous and mixed forests with deciduous stands of different age classes (including clearcuts) interspersed by lakes and bogs (Martin et al., 2010). The main tree species are Scots pine (*Pinus sylvestris*), Norway spruce (*Picea abies*), and birch (*Betula* spp.) with an abundant underlayer of berry shrubs, especially *Vaccinium* spp. (Elfström et al., 2008; Ordiz et al., 2013).

**FIGURE 1:**
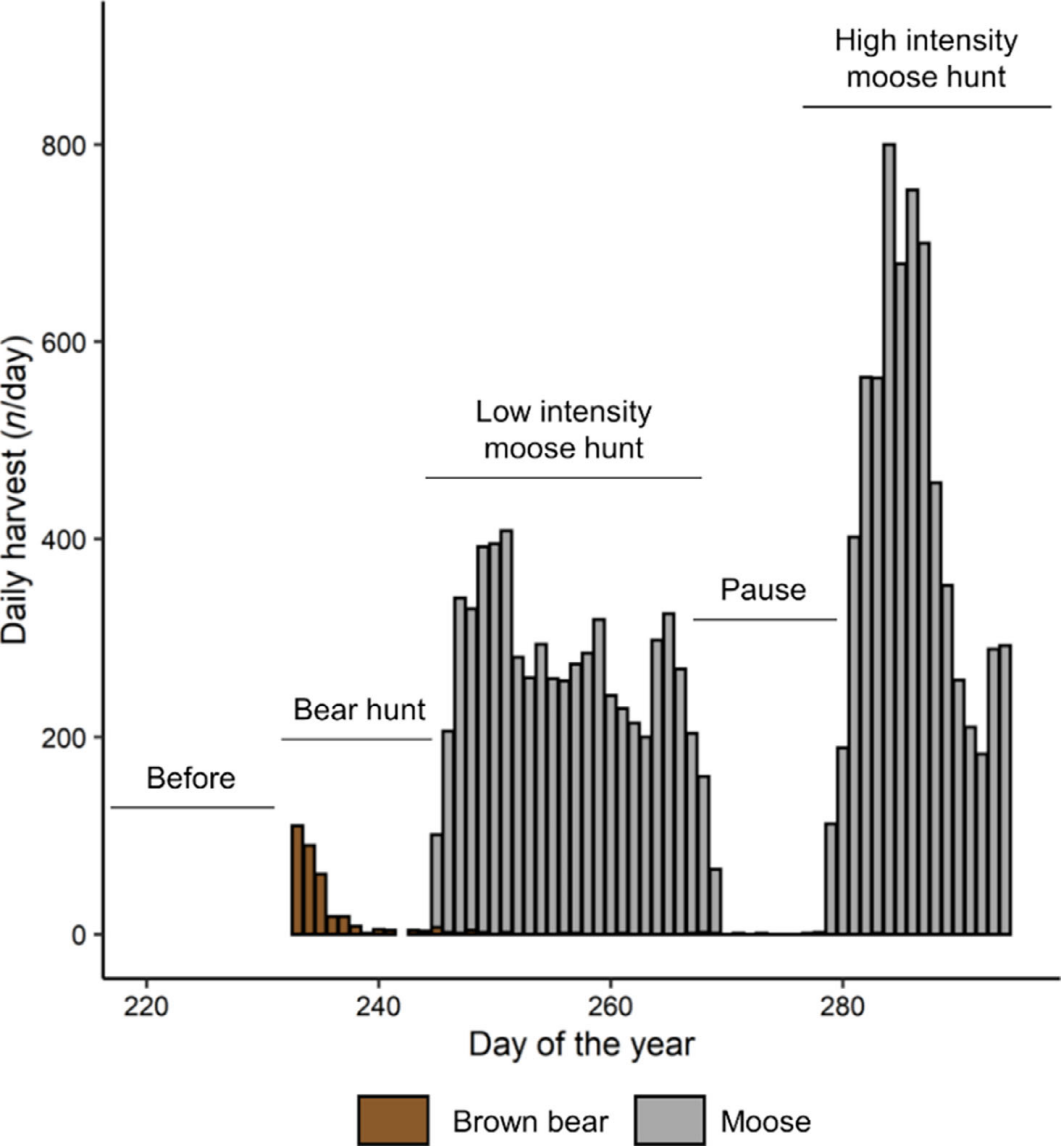
Number of moose and brown bears harvested per day of the year within moose management units that could have been used by GPS‐collared brown bears in south‐central Sweden, 2016–2019. Labels indicate how the fall was split into five periods based on bear and moose hunting patterns.

The bear hunting season in Sweden starts on 21 August and lasts until 15 October or until the regional quotas are filled, whichever comes first (Bischof et al., 2008), but most bears are shot within the first 3 days of the hunting period (Figure 1). Brown bears are hunted mainly using baying or pursuing dogs that pick up scent trails on roads or near bait sites (le Grand et al., 2019). All bears can be legally shot with the exception of family groups, that is, a female accompanied by dependent offspring of any age (Van de Walle et al., 2018).

The moose hunting season in our study area starts on the first Monday of September and lasts until February the next year; however, 94% of the moose harvest occurs from September through November (Wikenros et al., 2013). Between 2016 and 2019, only 6% of the 351 legally killed bears were harvested after the onset of moose hunting in our study area, indicating little overlap between bear and moose hunting seasons. In Sweden, moose are usually hunted with baying dogs that push them toward the locations of still hunters (Liberg et al., 2010; Sand et al., 2006). Our study area is divided into 17 moose management units (Appendix S1: Figure S1) and the Swedish County Administrations compile daily moose harvest data (number of harvested moose/day) during the hunting season for each unit. Moose hunting generally starts slowly in September and stops completely from the end of September to mid‐October to minimize disturbance during the peak of the moose rut (Figure 1). Then, moose hunting resumes at high intensity from mid‐October to early November (Figure 1). Based on the dates of the bear and moose hunting seasons, we split the fall into five periods for each year between 2016 and 2019: (1) we defined the period between 10 August and 20 August as “*before hunting*,” (2) the period between 21 August and the first Monday of September as “*bear hunt*,” (3) the period between the first Monday of September and the day of the last moose harvest in September as “*low‐intensity moose hunt*,” (4) the period between the day of last moose harvest in September and day of the first moose harvest in October as “*moose hunt pause*,” and (5) the day of the first moose harvest in October until 21 October as the period of “*high‐intensity moose hunt*” (see also Figure 1). We used 21 October as the final cut‐off, because most bears start to hibernate at this time (Friebe et al., 2014).

### Capture and handling

Between 2016 and 2019, female brown bears (*n* = 30 different bears; *n* = 53 bear‐years) were darted from a helicopter with a remote drug delivery system (Dan‐Inject, Børkop, Denmark). In this study, we excluded males because only two individuals were monitored; see Arnemo and Evans (2017) for more details on the capture protocol. Brown bears were equipped with GPS‐GSM collars (GPS Plus; Vectronic Aerospace, Berlin, Germany). All capture and handling protocols were approved by the Swedish Ethical Committee on Animal Research, Uppsala (C18/15) and the Swedish Environmental Protection Agency (NV‐00741‐18, NV‐01758‐14).

### Resource selection by moose hunters

Between 2016 and 2019, we obtained 3136 moose harvest locations from four moose management areas (Söder‐Voxnan, Voxna, Härjedalen and Ljusnan‐Voxnan) located in south‐central Sweden (Appendix S1: Figure S1). These locations were voluntarily provided by hunters, while registering killed moose on the official database of the Swedish County Administrations (Länsstyrelserna, 2019). Other management areas within our study area were not used by brown bears or few hunters disclosed moose harvest locations (resulting in poor coverage) and were therefore excluded from further analyses. Moose harvest data can be downloaded on Älgdata by selecting “ÄFO” under “Biologiskt data för fällda älgar” in the “Statistik” menu. Then, select either Gävleborgs, Dalarnas or Jämtlands under “Län” and search for the management units highlighted in yellow in Appendix S1: Figure S1 for each year between 2016 and 2019.

We used resource selection functions (RSF) to predict resource selection in moose hunters (Manly et al., 2002). We generated the same number of random positions (coded 0) as used moose harvest locations (coded 1) within each management area (in yellow; Appendix S1: Figure S1). We extracted the percentages of landcover types within buffers of 65 m radius centered on both moose harvest and random locations, which corresponds approximatively to the average distance moose are shot in Scandinavia (Stokke et al., 2017). Landcover types were extracted from a reclassified 10 × 10 m landcover map of the Swedish Environmental Protection Agency (Naturvårdsverket, 2018). Clearcut data were obtained from the Swedish Forestry Agency (Skogsstyrelsen, 2020) by entering “Utförda avverkningar” in the search box. We extracted the distance to the closest road (in meters) from a distance raster (10 × 10 m) based on the National Road Data Base of the Swedish Transport Administration (Trafikverket, 2019), which can be obtained by searching “Nationell vägdatabas” in the search box. Elevation was extracted from a digital elevation model (Lantmäteriet, 2004), which can be obtained by entering “Terrain Model Download, grid 50+” in the search box. The terrain ruggedness index (TRI) was derived from the digital elevation model using the TRI tool in QGIS version 3.14.0 (QGIS Development Team, 2020).

We built the RSF by using a generalized linear model with a binomial family and a logit link function (“glm” function, *stats* package; [R Core Team, 2021]). The model included variables known to influence visibility and accessibility within the landscape. We included the distance to the closest road, terrain ruggedness (with a quadratic term), elevation (with a quadratic term), the proportion of human infrastructure, the proportion of open bogs and clearcut and forest composition (young forest, 5–15 m conifer forest, 5–15 m mixed forest and 5–15 m deciduous forest). We used corrected Akaike information criterion (AIC_c_) to validate the choice of model (Appendix S1: Section S3). We used *k*‐fold cross‐validation to assess the predictive ability of our model (Boyce et al., 2002).

### Brown bear response to moose hunting

Our dataset contained a total of 82,042 bear locations recorded at 1‐h intervals between 10 August and 21 October 2016–2019. We filtered the data to only include locations with a dilution of precision <10 (D'Eon & Delparte, 2005) and removed lines with missing observations. For each location, we noted the moose management unit as well as the hunting period during which it was recorded: *before hunting*, *bear hunt*, *low‐intensity moose hunt*, *moose hunt pause*, or *high‐intensity moose hunt*.

We first used integrated step‐selection functions (iSSF) to determine how brown bears responded to moose hunting. The purpose of this analysis was not to build a model predicting resource selection in brown bears during the fall, but rather to use iSSF to directly model the bears' response to moose hunting. The iSSF approach consists in comparing a pair of consecutive GPS positions, referred to as a step (coded as 1), to a series of available steps (coded as 0) that are randomly generated around the starting location of the observed steps (Avgar et al., 2016; Thurfjell et al., 2014). We used the *amt* package (Signer et al., 2019) to generate 10 available steps for each observed step. The step length (in meters) and turning angle (in radian) of available steps were modeled from Gamma and von Mises distributions, respectively (Signer et al., 2019). We added 0.001 to all step lengths to avoid an error when log‐transforming steps with no movement (step length = 0 m). We extracted the mean relative probability of moose kill within 50 m at the end of each used and available steps and we included this variable with its quadratic term, the log of step length and the cosine of the turning angle as variables in conditional logistic regressions. We used the Poisson formulation of conditional logistic regressions fitted with *glmmTMB* (Brooks et al., 2017) as suggested by Muff et al. (2020) to account for interindividual differences in behavior and functional response. This approach requires adding the step ID as a random intercept and fixing its variance to a large value (i.e., 10^6^) and it allowed us to include random coefficients for all the variables in the models, thereby accounting for functional response and interindividual differences in resource selection (Muff et al., 2020).

We built separate models for each demographic group (females with dependent offspring, *n* = 18; lone females, *n* = 17; and subadult females, *n* = 18) because age and reproductive status are known for influencing the response to human disturbances in brown bears (Lamb et al., 2020; Ordiz et al., 2012). For each demographic group, we built two sets of models for steps recorded during the day and night based on the hours of sunrise and sunset (i.e., when the sun is 6° above the horizon), because it is forbidden to hunt moose from sunset to 1 h before sunrise in our study area. We adjusted sunrise/sunset times by ±1 h to account for potential disturbance related to hunter travel in/out of posts, transportation, or search for wounded game, which may extend past legal hunting hours. We also modeled the bears' response to the probability of moose kill (by hunters) separately for each hunting period (Figure 1) because we expected the behavior of brown bears and their response to human disturbance to change over our study period, resulting in a total of 30 models. The effect of the probability of moose kill was modeled during the “*before hunting*” and “*bear hunt*” periods, even though moose hunting had not yet started, to provide contrast with the moose hunting periods. We also created post hoc models to specifically investigate the movement response of brown bears to moose hunting. The movement parameters (log of step length and cos of turning angle) were added in the models and interacted with the probability of moose kill and its quadratic term extracted at the start of each step with a random coefficient for all parameters, including interaction terms (Table 1). Due to convergence issues, we could not create separate models for each demographic group in the movement models and we have added the results of these models as supporting information (see Appendix S1: Section S5).

**TABLE 1:** Structure of models used to estimate moose hunter resource selection function (RSF) and the integrated step‐selection functions (iSSF) for brown bears in south‐central Sweden, 2016–2019.

Models	Structure
Hunter RSF	Distance to road + Elevation + Elevation^2^ + Ruggedness + Ruggedness^2^ + Infrastructure + Clearcut + Open bog + Young forest + 5–15 m conifer forest + 5–15 m deciduous forest + 5–15 m mixed forest
Brown bear iSSF (RSFhunt selection)	RSFhunt + RSFhunt^2^ + log_sl + cos_ta
Brown bear iSSF (RSFhunt movement)	log_sl + cos_ta + log_sl:RSFhunt + log_sl:RSFhunt^2^ + cos_ta:RSFhunt + cos_ta:RSFhunt^2^
Brown bear iSSF (Landscape)	Young forest + 5–15 m conifer forest + 5–15 m deciduous forest + 5–15 m mixed forest + Clearcut + Distance to road + Ruggedness + Open bog + log_sl + cos_ta

*Note*: A random coefficient (e.g., [0 + Young forest|bear‐year]) was added for each variable, including movement parameters, quadratic terms, and interactions, listed in brown bear iSSF models. All bear models also included random intercept with fixed large variance (i.e., 10^6^) for step ID. The effects of time of day, demographic groups and hunting periods were accounted for in separate models, except in the iSSF movement models in which demographic groups were combined. The moose hunter RSF did not include random effects.

We used the relative selection strength (RSS) to visualize the bears' response to the relative probability of moose kill (Avgar et al., 2017). This method estimates how likely an animal is to select a location (*x*_1_) relative to another location (*x*_2_). It is calculated by generating predictions of the relative probability of use at the two locations (*x*_1_ and *x*_2_), while holding all other variables constant (e.g., at their mean) except the variable for which we aim to visualize the effect size. At the first location (*x*_1_), movement parameters were held constant at their mean (Table 2), while the relative probability of moose kill varied across the range of possible values. At the second location (*x*_2_), all variables were held at their mean value (see Appendix S1: Table S1). The RSS was calculated during each hunting period and time of the day, thereby allowing us to visualize the effect of the relative probability of moose kill during each hunting period. The RSS was computed using the calculations outlined in Avgar et al. (2017).

**TABLE 2:** Habitat selection coefficients (β) with standard error (SE) and lower and upper boundaries of 95% confidence intervals for moose harvest locations in south‐central Sweden, 2016–2019.

Variable	β	SE	Lower	Upper
Intercept	0.062	0.036	−0.009	0.133
**% Infrastructure**	**−0.530**	**0.128**	**−0.781**	**−0.280**
% Young forest	−0.035	0.027	−0.089	0.018
% Open bog	0.032	0.031	−0.028	0.092
**% Clearcut**	**0.106**	**0.028**	**0.052**	**0.160**
**Distance to road**	**−0.443**	**0.044**	**−0.530**	**−0.356**
**Elevation**	**0.274**	**0.035**	**0.206**	**0.342**
**Elevation** ^**2**^	**−0.060**	**0.022**	**−0.103**	**−0.016**
**Terrain ruggedness**	**0.158**	**0.037**	**0.085**	**0.230**
**Terrain ruggedness** ^**2**^	**−0.035**	**0.011**	**−0.056**	**−0.013**
**% 5–15 m Conifer**	**−0.115**	**0.028**	**−0.170**	**−0.061**
% 5–15 m Deciduous	0.030	0.027	−0.022	0.082
% 5–15 m Mixed	−0.053	0.027	−0.105	0.000

*Note*: Percent infrastructure denotes the percentage of cover occupied by permanent artificial structures within the 65 m buffers centered on moose harvest and available locations. Bolded rows indicate significant effect on selection.

### Brown bear habitat selection during moose hunting

We also modeled resource selection on a fine temporal scale in brown bears using iSSF. We extracted landcover types within circular buffers (radius = 50 m) centered at the end location of each step. We conducted a scaling experiment to determine the appropriate buffer size and concluded that buffers with a 50 m radius were the most suitable (Appendix S1: Figure S2). We included most variables from the hunter RSF with movement parameters (i.e., log of step length and the cosine of the turning angle) in determining the influence of moose hunting on resource selection in brown bears. We added random coefficients for each variable and formulated mixed conditional logistic regressions for each period with *glmmTMB* (Brooks et al., 2017; Muff et al., 2020) as described in the previous section. We did not include the relative probability of moose kill in combination with other landscape covariates due to a lack of independence between this variable and the others (i.e., rho_road_ = 0.58).

All landscape covariates were scaled by subtracting their mean value and dividing the difference by their standard deviation (see Appendix S1: Table S1) to facilitate model convergence. We screened numerical variables with a Spearman correlation matrix (“cor” function; *stats* package [R Core Team, 2021]) and variance inflation factors (“vif” function; *car* package [Fox & Weisberg, 2019]) to detect multicollinearity. All variables had low correlation coefficients (∣rho∣ < 0.40) and variance inflation factors were low (VIF ≤ 1.17) indicating low multicollinearity within our set of variables. The distance to the closest road was multiplied by −1 in the iSSF models to facilitate result interpretation. The RSS was calculated as described above. We used AIC_c_ to validate the choice of model (Appendix S1: Section S3). All statistical analyses were conducted in R version 4.1.0 (R Core Team, 2021) and maps were processed in QGIS version 3.14.0 (QGIS Development Team, 2020).

## RESULTS

### Resource selection by moose hunters

The hunter RSF showed that moose hunters in Sweden were more likely to shoot moose in areas closer to roads and with a higher proportion of clearcuts (Table 2). Moose hunters were also more likely to shoot moose at intermediate elevations and terrain ruggedness values (Table 2). Moose were more likely to get shot in areas with a lower proportion of human infrastructure (Table 2). Moose hunters were less likely to shoot moose in areas with higher proportions of mixed and coniferous forests (Table 2), whereas the proportion of young forests, deciduous forests, and open bogs did not influence the relative probability of moose kill (Table 2).

**FIGURE 2:**
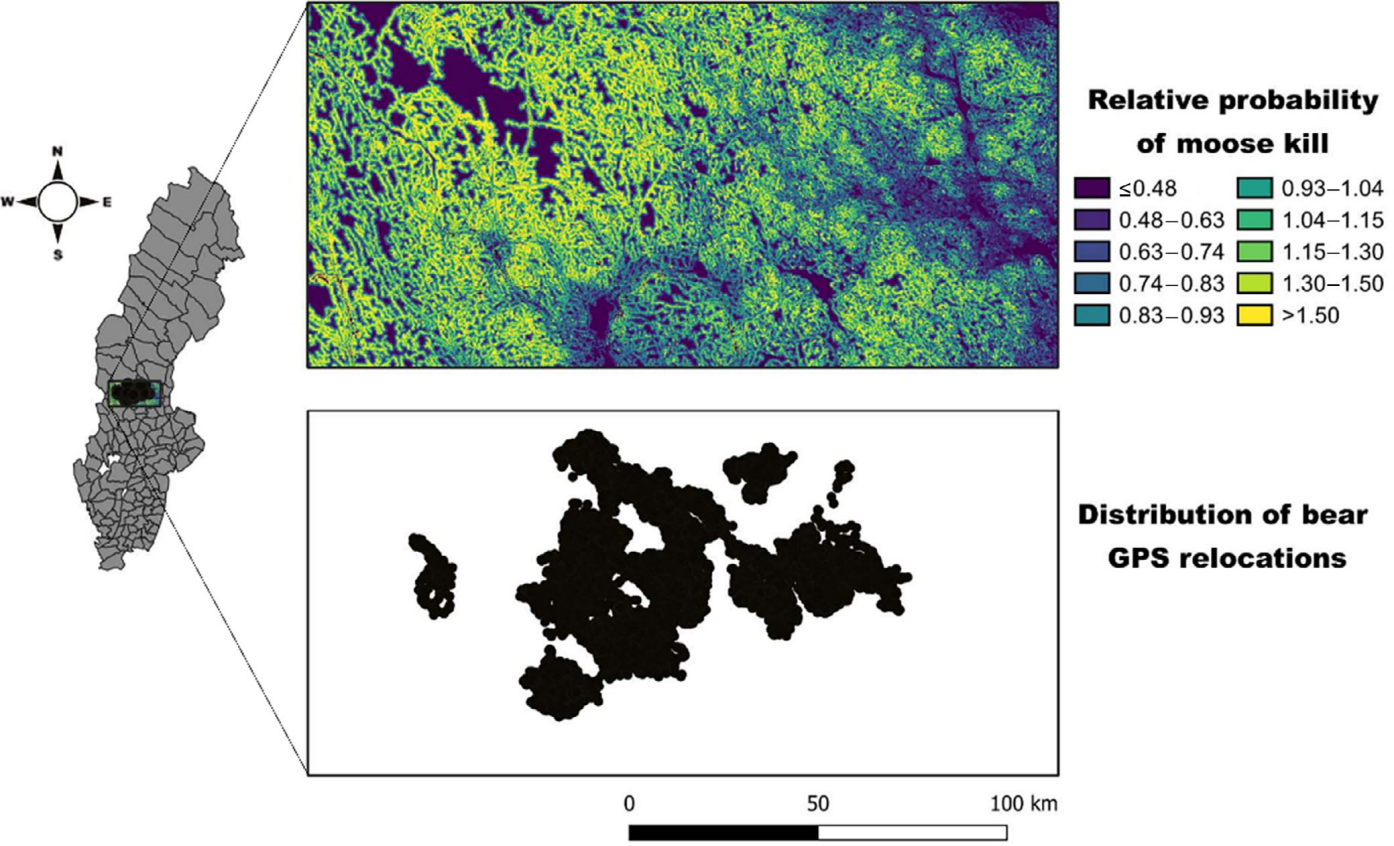
Relative probability of shooting a moose at a resolution of 100 m and the distribution of bear GPS relocations (*n* = 82,042 from 53 bear‐years) in south‐central Sweden during 2016–2019. The relative probabilities of moose kills were estimated from an exponential equation: *w*(*x*) = exp(β_1_ *x*_1_ + β_2_ *x*_2_ + … + β_*n*_ *x*_*n*_), where the β_*i*_ values were estimated from the full model (Appendix S1: Table S5). The 10 bins are based on 10% quantiles.

We assessed the predictive ability of our model with *k*‐fold cross‐validation by randomly dividing our dataset into five folds and ranking the RSF values within 10 quantile bins (Boyce et al., 2002; Roberts et al., 2017). The mean correlation between bin ranks and the adjusted area frequencies was *r*_s_ = 0.88 and ranged from 0.72 to 0.97, thereby indicating high predictive power (Boyce et al., 2002). Therefore, we used this model to calculate the relative probability of hunters harvesting a moose at a 100 m resolution across our study area (Figure 2).

### Brown bear response to moose hunting

**FIGURE 3:**
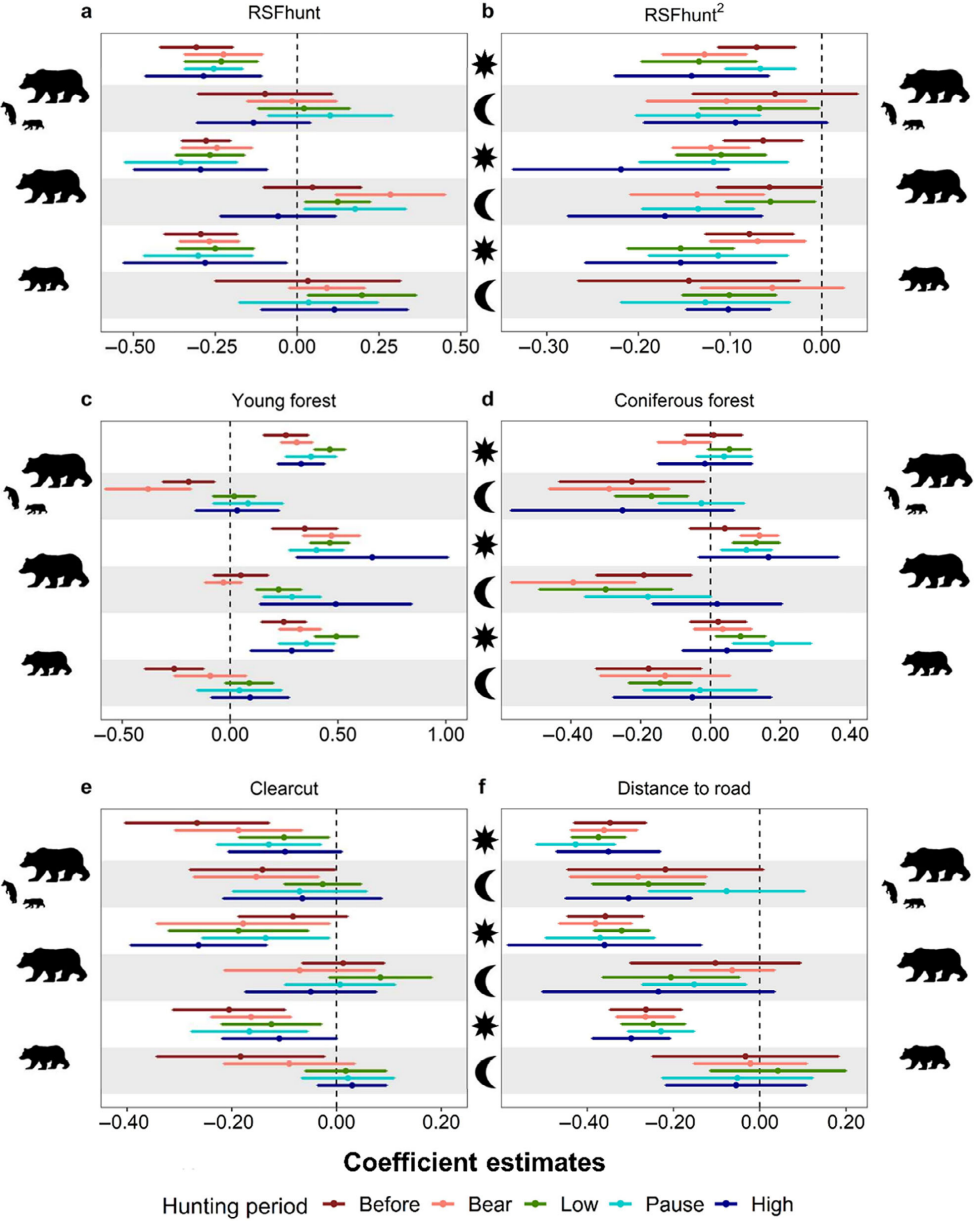
Coefficient estimates for the selection of probability of moose kill (a, RSFhunt), its quadratic term (b, RSFhunt^2^), (c) young forest, (d) coniferous forest, (e) clearcut, and the (f) distance to the closest road with 95% confidence intervals. The coefficients were estimated from integrated step‐selection functions (iSSF) for female brown bears with dependent offspring (*n* = 18 bear‐years), solitary females (*n* = 17 bear‐years) and subadult females (*n* = 18 bear‐years) for day and night in south‐central Sweden, 2016–2019. The coefficients were estimated for each hunting period: before hunting (red), bear hunt (pink), low‐intensity moose hunt (green), pause (cyan), and high‐intensity moose hunt (dark blue). Other parameters are presented in Appendix S1: Figures S3 and S4, Table S6.

**FIGURE 4:**
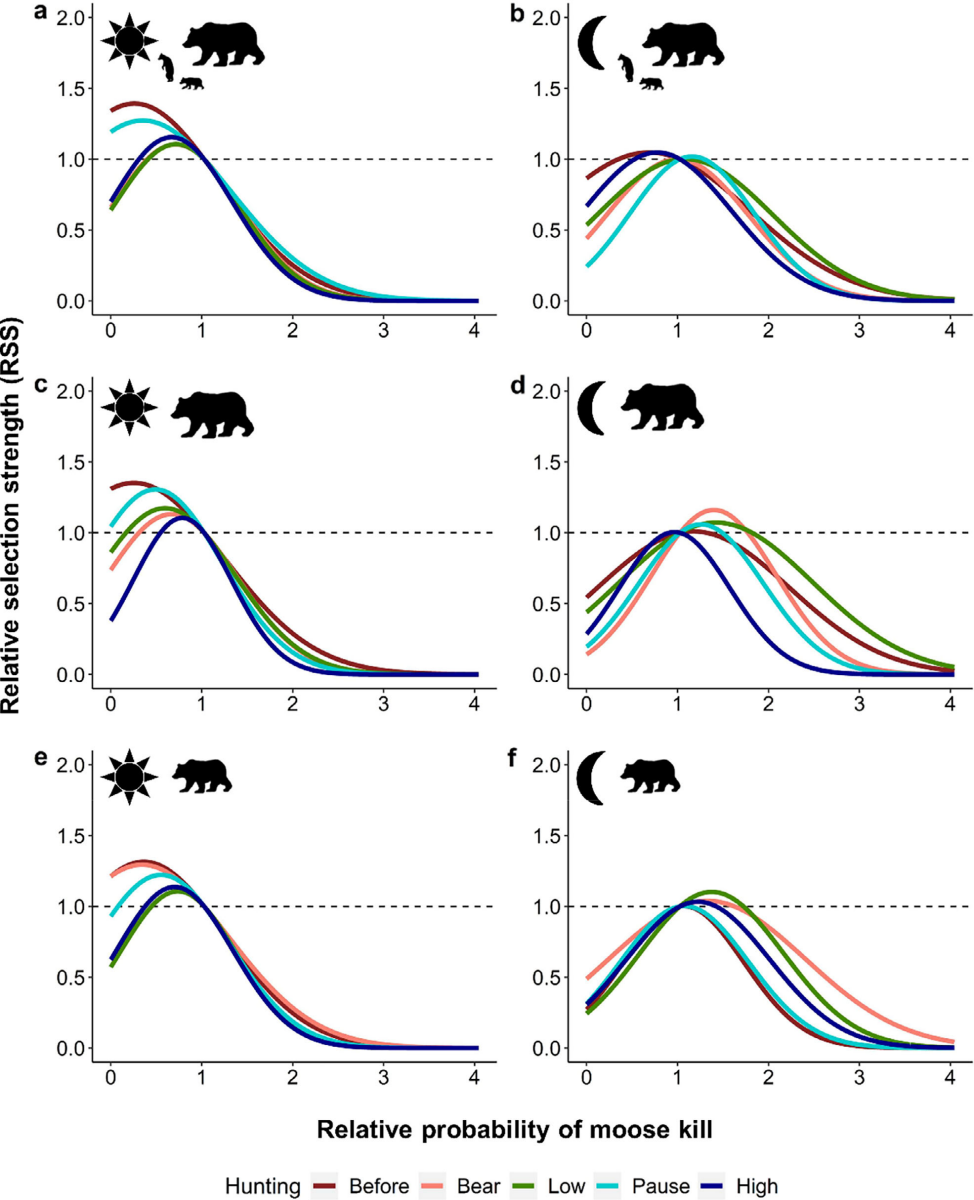
Relative selection strength (RSS) for the probability of moose kill (by hunters) in (a, b) female brown bears with dependent offspring (*n* = 18 bear‐years), (c, d) solitary females (*n* = 17 bear‐years) and (e, f) subadult females (*n* = 18 bear‐years) for day and night in south‐central Sweden, 2016–2019. The RSS shows how much likely a bear is to select a location relative to another location. An RSS of 1.5 at location *x*_1_ indicates that brown bears were 1.5 times more likely to use location *x*_1_ relative to location *x*_2_. The RSS was calculated for each hunting period: before hunting (red), bear hunt (pink), low‐intensity moose hunt (green), pause (cyan) and high‐intensity moose hunt (dark blue).

Brown bears generally avoided areas with a higher probability of moose kills, especially during the day (Figures 3 and 4). Females with dependent offspring weakly selected for areas with an intermediate probability of moose kills at night during the *low‐intensity moose hunt* and the *moose hunt pause* (Figures 3a,b and 4). Solitary females were also more likely to select areas with intermediate probability of moose kills at night *before hunting* and during *bear hunt*, the *low‐intensity moose hunt*, and the *moose hunt pause* (Figures 3a,b and 4). Subadult females selected areas with intermediate probability of moose kills at night during all hunting periods (Figures 3a,b and 4). Females with dependent offspring strongly avoided areas with a higher probability of moose kills during the daylight hours of both *bear* and *moose hunting* periods (Figure 3b). Bears from all demographic groups became gradually less active as the fall progressed and took shorter steps (i.e., traveled shorter distances) that were less directional, except females with dependent offspring that took longer and more directional steps (i.e., straight movements) during the daylight hours of the bear hunt (Appendix S1: Table S6 and Figure S4). Female brown bears also moved faster and more directionally when traveling in areas with a higher probability of moose kills (Appendix S1: Table S7 and Figure S5).

### Brown bear habitat selection during moose hunting

**FIGURE 5:**
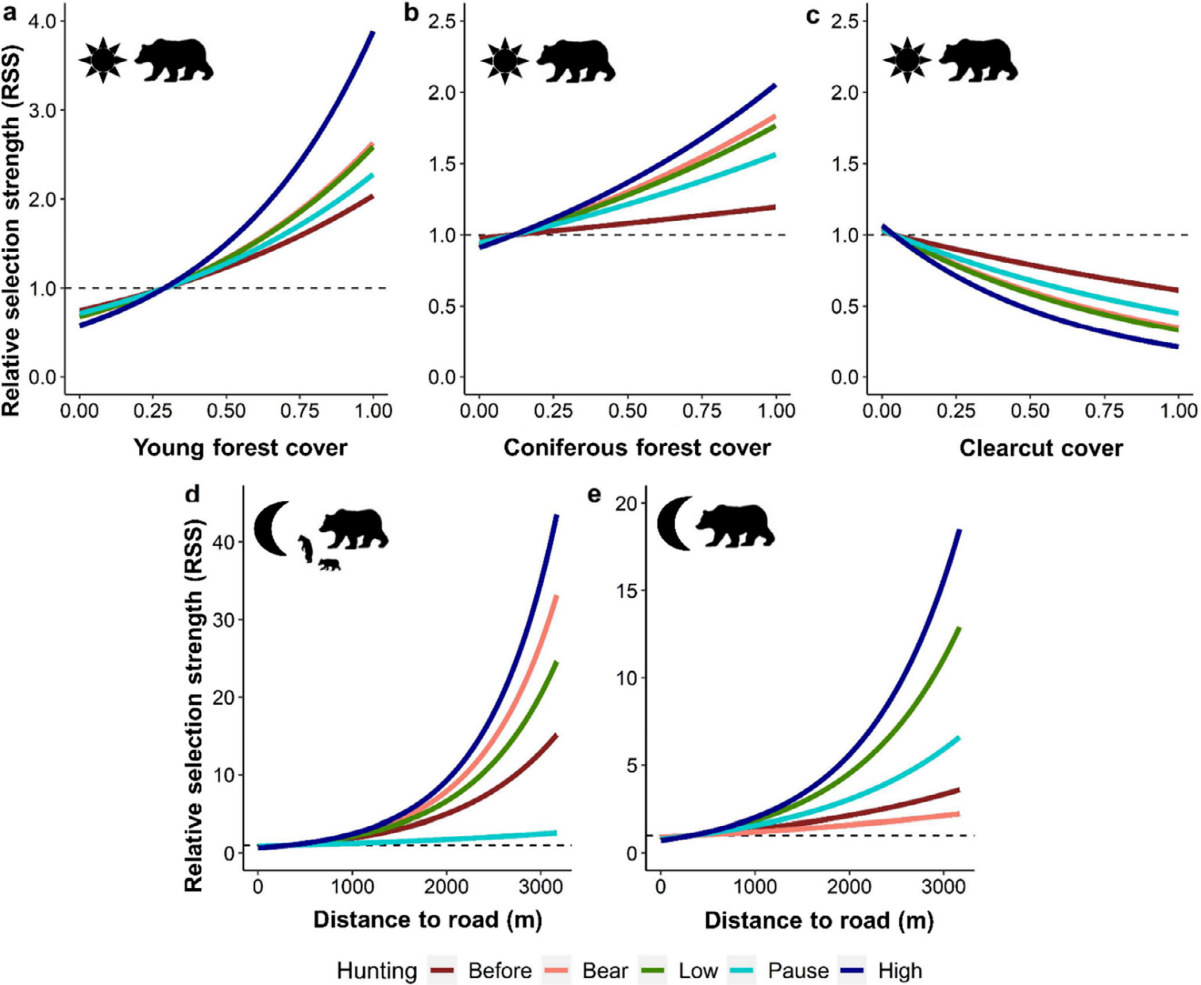
Relative selection strength (RSS) for (a) young forest by solitary females (*n* = 17 bear‐years) during the day, (b) coniferous forest by solitary females during the day, (c) clearcuts by solitary females during the day and (d) and (e) the distance to the closest road by females with dependent offspring (*n* = 18 bear‐years) and solitary females during the night in south‐central Sweden, 2016–2019. The RSS shows how much likely a bear is to select a location relative to another location. An RSS of 1.5 at location *x*_1_ indicates that brown bears were 1.5 more likely to use location *x*_1_ relative to location *x*_2_. The RSS was calculated for each hunting period: before hunting (red), bear hunt (pink), low‐intensity moose hunt (green), pause (cyan), and high‐intensity moose hunt (dark blue). Note the scale difference between panels (d) and (e).

Our results indicate that brown bear habitat selection varied substantially during the fall and some changes (i.e., selection of young forest, clearcuts and distance to the closest road) were consistent with disturbances associated with bear and moose hunting (Figures 3 and 5). Brown bears selected for young forests during the day of all hunting periods (Figures 3c and 5a). The diurnal use of young forests by females with dependent offspring and subadults peaked during the *low‐intensity moose hunt*, whereas it was highest during the *bear hunt* and both periods of *moose hunting* in solitary females (Figure 3c). Solitary females were also more likely to select areas with higher proportions of coniferous forest during the days of the *bear hunt* and during *low‐* and *high‐intensity moose hunt* compared with the other periods (Figures 3d and 5b).

Areas with higher proportions of clearcut were generally avoided during the day, whereas those areas were less strongly avoided or used in proportion to their availability at night (Figure 3e). Females with dependent offspring and subadults slightly increased the diurnal use of clearcuts during the periods of *low‐* and *high‐intensity moose hunt*, whereas solitary females showed the strongest avoidance of clearcuts during the *bear hunt* and both *moose hunting* periods (Figure 3e).

Bears from all demographic groups avoided roads during the days of all periods, whereas roads were less strongly avoided during the night (Figure 3f). The selection coefficients for roads during the day did not vary much across hunting seasons in all demographic groups (Figure 3f). During the night of hunting periods the selection coefficient for adult females was closer to coefficient estimates during the day, whereas such changes were not evident in subadults (Figures 3f and 5d,e). Roads were avoided more strongly by solitary females at night during both periods of *moose hunting*. A similar pattern was observed in females with dependent offspring, but they also avoided roads more strongly at night during the *bear hunt* (Figures 3f and 5d,e). The other parameters did not show substantial variations according to hunting periods and are reported in the supporting information (Appendix S1: Figure S3 and Table S6).

## DISCUSSION

In this study, we found that the activity patterns of hunters and their use of the landscape influenced habitat selection by female brown bears. Specifically, we investigated if and how female brown bears react to moose hunting because moose hunters can alter the availability of food resources as well as create a landscape of fear. Our analyses supported our a priori hypothesis that moose kills are located closer to roads and in areas with good visibility. Our results also suggest that female brown bears perceive moose hunting activities as a threat because they generally avoided areas with a high probability of moose kills. Solitary female bears increased the selection for better concealment during the daytime hours of both the *bear* and *moose hunt*. We additionally found that females avoided roads more strongly during the night in both the *bear* and the *moose hunting periods*. We did not find strong support for the prediction that, during the night, female brown bears were attracted to areas with high probability of moose kills. Overall, our results suggest that moose hunting was perceived as a threat and created a landscape of fear, and that slaughter remains were not strong attractants for female bears in south‐central Sweden.

Moose harvest locations were not randomly distributed across the landscape and as expected, were concentrated around specific landscape features that favored accessibility and visibility such as proximity to a road, high proportions of clearcuts and intermediate values of elevation and terrain ruggedness. This pattern was expected because the road network allows hunters to easily move across the landscape and it has been documented in multiple systems regardless of the targeted species (Boer, 1990; Ciuti et al., 2012; Lebel et al., 2012; Leclerc et al., 2019; Paton et al., 2017). Rugged terrain and/or high elevations may also impede the movement of hunters across the landscape and restrict access (Diefenbach et al., 2005); however, slightly elevated and mid‐slope locations could improve visibility by providing hunters with better vantage points and more open shooting lanes. Our study showed that visibility influenced the distribution of moose harvest locations, which is supported by moose harvest locations being disproportionally distributed in areas with higher proportions of clearcuts. The importance of visibility is more disputed in the literature (Perry et al., 2020) and ultimately depends on the hunting tactics (i.e., passive vs. active) used by hunters (Norum et al., 2015). Animals can perceive predators as a threat and in response allocate more time to antipredator behaviors (Lima & Bednekoff, 1999); however, spending more time hiding often comes at the expense of foraging opportunities and can carry substantial costs (Hertel, Zedrosser, et al., 2016; Lima & Dill, 1990). Solitary females responded to variations in perceived risk from moose hunting activities by increasing the selection of landcover classes with short to medium conifers (i.e., young and coniferous forests) that obstruct lateral visibility. Selection for better concealment during the hunting season is a common antipredator response that has been reported in bears and other game species (Gaynor et al., 2022; Lone et al., 2015; Ordiz et al., 2011; Paton et al., 2017; Thurfjell et al., 2013), but to our knowledge, such a response has rarely been documented in a large carnivore that is not specifically targeted by hunters (Dobbins et al., 2020). Previous work has shown that brown bears in Sweden forage less efficiently during the bear hunt, which may negatively affect their body condition (Hertel, Zedrosser, et al., 2016). Our results suggest that bears were unable to differentiate between bear and moose hunters, likely because they use similar hunting methods, and thus responded similarly during both hunting seasons. Therefore, we can expect the foraging efficiency of bears to be negatively affected during moose hunting. Consecutive and partially overlapping hunting seasons in the fall likely introduce continuous disturbance during the last weeks of hyperphagia in bears. It is important to consider that our analyses were based on successful moose harvest locations and that all other hunting activities were not included in the model, which suggests that we likely underestimated the disturbance associated with moose hunting.

The disturbances induced by human activities are heterogeneously distributed in space and time (Gaynor et al., 2022). For instance, humans tend to concentrate their activities in areas closer to roads and stronger avoidance of these areas has been documented in several large mammals (Bonnot et al., 2013; Ciuti et al., 2012; Ladle et al., 2019; Paton et al., 2017). Our results show that brown bears in Sweden have a similar response. We observed the largest differences in selection for areas close to roads between hunting periods in females with offspring and solitary females during the night; however, we expected such behavioral changes to occur during the day when hunters are active (Brown et al., 2020; Gaynor et al., 2018; Paton et al., 2017). Brown bears already avoided roads during the day before the onset of hunting and because roads are a network of interconnected linear features at relatively high density in our study area, it might be impossible for bears to move further away from a road without moving closer to another one, thereby explaining the lack of variation in road selection across hunting periods during the day. Following an encounter with humans, bears change both their day and nighttime movement behavior and activity pattern for up to 2 days after the disturbance (Ordiz et al., 2013). Thus, the pronounced changes in road selection during the night could be carry‐over effects from daytime disturbances. This suggests that the effects of human disturbances are long‐lasting in bears, and it would also explain why slaughter remains discarded by hunters did not seem to be strong attractants in our study. Long‐lasting effects of bear hunting during the moose hunting periods cannot be completely excluded but are unlikely a limitation in this study because experimental approaches simulating human disturbance have shown that the behavior of Scandinavian brown bears returns to normal within 3 days following the disturbance (Ordiz et al., 2013). Therefore, it is unlikely that a short period of intensive hunting caused behavioral changes over several months. Disturbances from bear hunting or seasonal variations would also not account for changes in resource selection that occurred during the *moose hunt pause* as the habitat selection coefficients for this period were similar to those estimated for *before hunting*, which suggests that moose hunting indeed caused some of the observed changes.

There is no selective advantage for an opportunistic scavenger to avoid highly nutritious food resources such as slaughter remains (DeVault et al., 2003), which suggests that this response is likely related to fear induced by human activities. Animals develop stronger antipredator responses when exposed to higher levels of human activity (Dobbins et al., 2020; Lamb et al., 2020). Thus, we can expect that bears in the hunted population living in an anthropized landscape to become wary of humans and avoid high‐risk areas. Previous studies showed that bears are able to identify low‐risk–high‐reward areas (Lodberg‐Holm et al., 2019), while feeding on berries to build up fat reserves (Hertel et al., 2018). Consequently, we expect bears in Sweden to feed sufficiently without the need to take additional risks to obtain slaughter remains; however, other carnivores that are more reliant on animal proteins could take more risks and approach humans to access food resources (Blecha et al., 2018). If a similar study was conducted across a gradient of anthropogenic disturbances, we would expect individuals from less disturbed landscapes to be less afraid of hunters, thereby affecting the balance of perceived risks and benefits.

Brown bears exhibited changes in resource selection during the fall that could not be easily attributed to moose hunting and we also observed marked differences across demographic groups. For example, the pattern of young forest selection in solitary females differed from that of subadults and females with dependent offspring. The reason for these different patterns of resource selection is unclear, but family groups are protected in Sweden (Van de Walle et al., 2018), which may alter their perception of potential risk. However, it may be an unlikely explanation because risk perception is similar in bears from all demographic groups when experimentally approached by humans (Ordiz et al., 2013). Alternatively, we would expect females with dependent offspring and subadults to take more risk later during the fall to maintain optimal foraging efficiency due to higher energy requirements during lactation (López‐Alfaro et al., 2013) and greater thermoregulatory costs in smaller individuals (Humphries et al., 2003; Manchi & Swenson, 2005). As berry patches become more depleted during the fall (Hertel, Steyaert, et al., 2016), females with dependent offspring and subadults may also search for alternative food sources such as ants (Frank et al., 2015) or slaughter remains in clearcuts, resulting in resource selection patterns that differ from that of solitary females. Another explanation, and potential limitation, of our study is that the presence of large males around slaughter remains could deter females from using these locations, but we could not investigate this aspect due to the low sample size of males. There is evidence from Sweden that females with dependent offspring avoid slaughter remains due to the presence of dominant conspecifics at these sites (Elfström et al., 2014). The results of our study, however, suggest that all three female demographic groups avoided areas with a higher probability of moose kills. Data from camera trap surveys also showed that brown bears, in general, were not commonly observed at hunter kill sites in Scandinavia (Gomo et al., 2017; Wikenros et al., 2013). Although, these surveys were conducted in areas with low bear densities, they support the contention that bears generally perceive moose hunters as a threat because if slaughter remains were strong attractants, we would expect bears to scavenge on them even at low bear densities.

## CONCLUSION

Our study found within‐season variation in brown bear habitat selection and showed that this variation is affected by the activity patterns of hunters and their use of the landscape. Ultimately, predation as well as human harvest has a multitude of effects on the behavior of targeted prey species (e.g., increased vigilance [Paton et al., 2017], indirect habitat loss [Dwinnell et al., 2019], nutritional costs [Hertel, Zedrosser, et al., 2016]); however, our results show that hunting also can trigger antipredator responses in nontarget species. The potential consequences of ungulate hunting on the behavior of bears might have remained unnoticed if our analyses had not accounted for temporal variations in ungulate hunting intensity. The costs associated with a landscape of fear can be substantial for wildlife (Dwinnell et al., 2019; Hertel, Zedrosser, et al., 2016) and they should be considered when planning and managing hunting seasons. Managers may wish to plan different hunting seasons simultaneously to concentrate the disturbance induced by hunting activities within a shorter timeframe. Our study also highlights the importance of collecting both temporal and spatial data on harvested wildlife as they can be used to model a landscape of fear, thereby providing valuable insights into the effects of human activities on wildlife. We recommend that future studies investigating the effects of human disturbances consider carrying out their analyses at scales reflecting temporal changes in risk.

## CONFLICT OF INTEREST STATEMENT

The authors declare no conflicts of interest.

## Supporting information


Appendix S1.

## Data Availability

Data used in habitat selection analyses (hunters and bears) can be found on Dryad in Brown et al., 2023 at https://doi.org/10.5061/dryad.sxksn037d.
