# Supplementary material for: Landscape of fear or landscape of food? Moose hunting triggers an antipredator response in brown bears
Source: Ecol Appl. 2023 Mar 26;33(4):e2840. doi: 10.1002/eap.2840 (PMC10909462; doi:10.1002/eap.2840)
Supplement: Supplementary file 1 — Appendix S1. [file EAP-33-e2840-s001.pdf]

Appendix S1

Supplementary material for: Landscape of fear or landscape of food? Moose hunting triggers an antipredator response in brown bears

Authors: Ludovick Brown, Andreas Zedrosser, Jon M. Arnemo, Boris Fuchs, Jonas Kindberg, Fanie Pelletier

Section S1: Background information

Section S2: Scaling experiment for resource selection in brown bears

Section S3: Model selection hunter RSF and bear iSSF

Section S4: Complementary results

Section S5: Post-hoc movement iSSF

Section S1: Background information

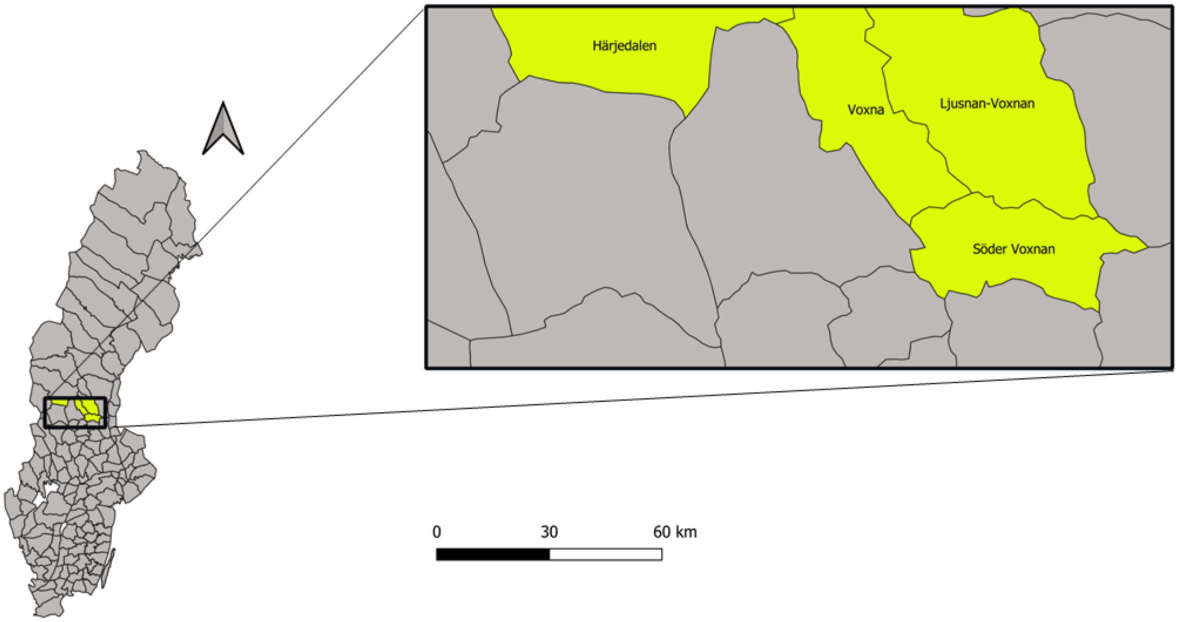

Figure S1. Map of the moose management units in Sweden. The rectangle represents the study area where brown bears were marked in south-central Sweden. The yellow zones (Härjedalen, Voxna, Ljusnan-Voxnan and Söder Voxnan) represent the moose management units from which we extracted moose harvest locations, 2016-2019.

Table S1. Variables included in integrated step-selection functions of brown bears (n = 53) during the fall in south-central Sweden, 2016-2019.

Variable	Description	Mean ± SD
Used	Available (0) and used (1) steps	-
Young forest (< 5m)	% forest < 5m tall within 50m-buffer	28.7 ± 34.6
Conifer forest (5-15m)	% coniferous forest (5-15m tall) within 50m-buffer	11.7 ± 20.3
Mixed forest (5-15m)	% mixed forest (5-15m tall) within 50m-buffer	2.44 ± 7.85
Deciduous forest (5-15m)	% deciduous forest (5-15m tall) within 50m-buffer	0.96 ± 4.87
Clearcut	% clearcut within 50m-buffer	3.94 ± 16.2
Open bog	% treeless bog within 50m-buffer	4.73 ± 13.9
Distance to road (m)	Distance to the closest road	318 ± 229
Terrain ruggedness	Terrain ruggedness index	11.1 ± 8.33
Step length [log(m)]	Log of distance travelled in 1h	4.25 ± 2.35
Turning angle [cos(rad)]	Cosine of movement direction	0.08 ± 0.71
RSFhunt	Relative probability of moose kill by hunters	1.03 ± 0.36
Hunting	Hunting periods (five level factor)	-

Notes: The mean ± standard deviation (SD) represent raw landscape features extracted from buffers (radius = 50m) centered at the end of used and available steps. Step lengths and turning angles are log- and cosine-transformed, respectively. Original Nationella marktäckedata classes were 118 and 128 for young forest, 111 to 113 for coniferous forest, 114 for mixed forest, 115 to 117 for deciduous forest and 2 for open bog.

Section S2: Scaling experiment for resource selection in brown bears

As resource selection is scale-dependent in large carnivores (Pitman et al. 2017), we needed to identify the scale at which landcover types should be extracted. To choose the appropriate scale, we conducted a scaling experiment, whereby landcover types were extracted within buffers of various radii [0m (dummy coded), 50m, 100m, 150m, 250m, 350m] centered on the end location of each step. We used the *fit_issf* function from the *amt* package to fit conditional logistic regressions with step ID as strata to each bear-year individually (Signer et al. 2019). We used AICc, as described in the main document, to compare the performance of the following model structure: Used (0,1) ~ % Deciduous (5-15m) + % Conifer (5-15m) + % Young forest (< 5m, >7 years old) + % Bog + % Clearcut (< 7 years old) + Distance to Road + Terrain ruggedness + log(step length) + cos(turning angle), with datasets that differed only in the scale (i.e., buffer size) at which landcover types were extracted. We counted the number of bear-years for which each scale explained the most variance and our results suggest that most bears selected resources at the 50m scale (Fig. S2). We consequently used this dataset in further analyses.

The dummy model returned a warning regarding a potential infinite Beta for the Clearcut class in a single individual; however, that model was not the top-ranked within the set and it did not influence the results of the scaling experiment.

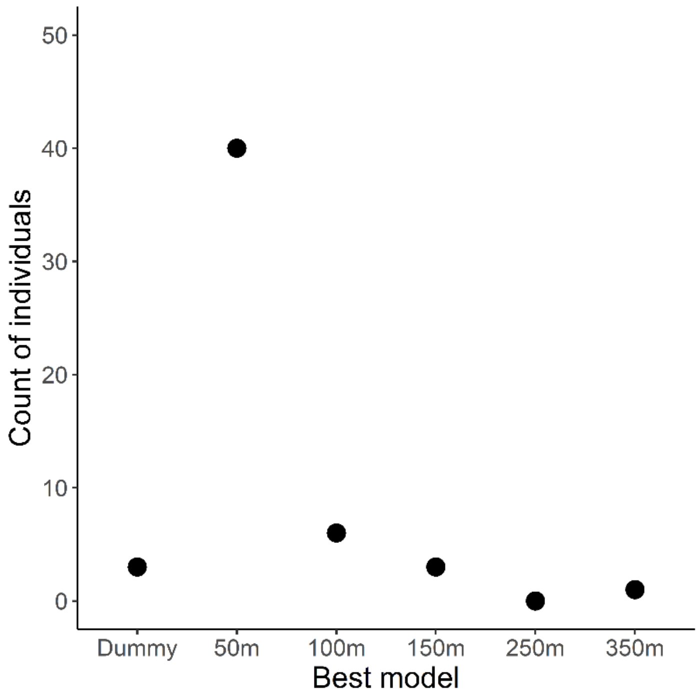

Figure S2. The number of individuals (n = 53 bear-years, in south-central Sweden, during 2016-2019) for which each scale explained the most variance. The models were named after the buffer sizes (Dummy = 0m radius) and differed only in the scales from which landcover types were extracted. Model selection was conducted by AICc.

Section 3: Model selection hunter RSF and bear iSSF

We built seven candidate generalized linear models with a binomial family and a logit link function [*glm* function, *stats* package; (R Core Team 2021)] for the resource selection by moose hunters. The seven models represented competing hypotheses: 1) that the probability of moose kills is random or that it depends on 2) accessibility only (Distance to road, elevation, terrain ruggedness, proportion of human infrastructures), 3) the proportion of open areas (clearcut and open bogs), 4) accessibility and the proportion of open areas, 5) forest composition (Young forest <5m, >7 years old; 5-15m conifer forest; 5-15m mixed forest and 5-15m deciduous forest), 6) forest composition and accessibility and 7) accessibility, the proportion of open areas and forest composition combined (Table S2). Model selection was conducted using second order Akaike Information Criterion (AICc) and its derived measures (ΔAICc and Akaike weight) from the AICc*modavg* package (Mazerolle 2020). Models within ΔAICc < 2 were considered equivalent (Burnham and Anderson 2002).

Table S2. Structure of candidate models used to estimate habitat selection coefficients for moose hunters in south-central Sweden, 2016-2019.

Models	Structure
1. Null	Used ~ 1
2. Accessibility	Used ~ Distance to road + Elevation + Elevation^2 + Terrain ruggedness + Terrain ruggedness^2 + Infrastructure
3. Open	Used ~ Clearcut + Open bog
4. Open + accessibility	Used ~ Distance to road + Elevation + Elevation^2 + Terrain ruggedness + Terrain ruggedness^2 + Infrastructure + Clearcut + Open bog
5. Forest composition	Used ~ Young forest + 5-15m conifer forest + 5-15m deciduous forest + 5-15m mixed forest
6. Forest composition + accessibility	Used ~ Distance to road + Elevation + Elevation^2 + Terrain ruggedness + Terrain ruggedness^2 + Infrastructure + Young forest + 5-15m conifer forest + 5-15m deciduous forest + 5-15m mixed forest
7. Full model	Used ~ Distance to road + Elevation + Elevation^2 + Terrain ruggedness + Terrain ruggedness^2 + Infrastructure + Clearcut + Open bog + Young forest + 5-15m conifer forest + 5-15m deciduous forest + 5-15m mixed forest

Notes: The model names reflect their underlying hypothesis. All models were fitted using generalized linear models with a binomial family and logit link function. The original Nationella Marktäckedata classes were 51 and 52 for the variable infrastructure. See table S1 for the other variables.

We also modelled resource selection at fine temporal scale in brown bears by using iSSF. To determine the best fixed effect structure, we first fitted conditional logistic regressions with step ID as strata by using the *fit_issf* function for each demographic group and for both day and night, separately (Signer et al. 2019). Each set contained three models built with a different set of variables and represented competing hypotheses (Table S3). In the Open model, brown bear

habitat selection depends only on open habitats and included variables were clearcut, distance to road and open bog. In the second model (Natural model), brown bear habitat selection depends only on forest composition (i.e., young, deciduous, mixed and coniferous forests) and terrain ruggedness. The third model included the variables from both the Natural and Open models. We could not include the relative probability of moose kill in combination with landscape covariates due to lack of independence between this variable and the others. We added the log of step length and the cosine of the turning angle and an interaction between each covariate and the hunting period in all candidate models.

Table S3. Structure of candidate models used to estimate habitat selection coefficients for brown bears (*n* = 53) in south-central Sweden, 2016-2019.

Models	Structure
Open	Used ~ Clearcut/hunting + Open_bog/hunting + Distance to road/hunting + log(Step_length)/hunting + cos(Turning_angle)/hunting
Natural	Used ~ Young_forest/hunting + Coniferous/hunting + Mixed/hunting + Deciduous/hunting + Terrain ruggedness/hunting + log(Step_length)/hunting + cos(Turning_angle)/hunting
Natural + Open	Used ~ Distance to road/hunting + Clearcut/hunting + Open_bog/hunting + Young_forest/hunting + Coniferous/hunting + Mixed/hunting + Deciduous/hunting + Terrain ruggedness/hunting + log(Step_length)/hunting + cos(Turning_angle)/hunting

Notes: All models were fitted using conditional logistic regression with step ID as strata. '/hunting' denotes an interaction between the variables and the hunting periods defined in Fig. 1 from the main manuscript.

The best performing RSF model for moose hunter was the Full model, which had a ΔAICc of ≥11.48 to the next best model (Table S4) and was also attributed 100% of the weight within the model set (Table S4). The four best models all included the variables elevation, terrain ruggedness and distance to roads (Table S4).

Table S4. Model selection by Akaike Information Criterion (AICc) for candidate generalized linear models used to estimate habitat selection coefficients for moose hunters in south-central Sweden, 2016-2019.

**Models**	**K**	**AICc**	**ΔAICc**	***w***	**LL**
7. Full	13	8414.95	0	1	-4194.45
6. Forest composition + Accessibility	11	8426.43	11.48	0	-4202.19
4. Open + Accessibility	9	8430.19	15.24	0	-4206.08
2. Accessibility	7	8450.89	35.94	0	-4218.44
3. Open	3	8662.75	247.8	0	-4328.37
5. Forest composition	5	8692.38	277.43	0	-4341.18
1. Null	1	8696.84	281.89	0	-4347.42

Notes: K = number of parameters, ΔAICc is the difference with the lowest AICc value, *w* is the model weight and LL is the log-likelihood of the model.

For the bear iSSF, the combination of the natural and open models performed best for all demographic groups during both day and night, and this model structure was attributed 100% of the weight in all model sets (Table S5).

Table S5. Model selection by Akaike Information Criterion (AICc) for integrated step-selection models in brown bears during day and night in south-central Sweden, 2016-2019.

	Model	K	AICc	ΔAICc	*w*	LL
Females with offspring (Day)	Natural+Open	50	79073.22	0	1	-39486.60
	Natural	35	80284.62	1211.40	0	-40107.30
	Open	25	80581.27	1508.05	0	-40265.63
Females with offspring (Night)	Natural+Open	50	46609.50	0	1	-23254.73
	Natural	35	46968.65	359.15	0	-23449.32
	Open	25	47005.23	395.73	0	-23477.61
Solitary females (Day)	Natural+Open	50	74960.88	0	1	-37430.42
	Natural	35	75764.33	803.46	0	-37847.16
	Open	25	76608.09	1647.21	0	-38279.04
Solitary females (Night)	Natural+Open	50	43883.14	0	1	-21891.55
	Natural	35	44209.48	326.34	0	-22069.73
	Open	25	44344.51	461.37	0	-22147.25
Subadult females (Day)	Natural+Open	50	81825.99	0	1	-40862.98
	Natural	35	82480.75	654.76	0	-41205.37
	Open	25	83377.24	1551.25	0	-41663.61
Subadult females (Night)	Natural+Open	50	48904.62	0	1	-24402.29
	Open	25	49072.81	168.19	0	-24511.40
	Natural	35	49179.53	274.92	0	-24554.76

Notes: K = number of parameters, ΔAICc is the difference with the lowest AICc value, *w* is the model weight and LL is the log-likelihood.

Section 4: Complementary results

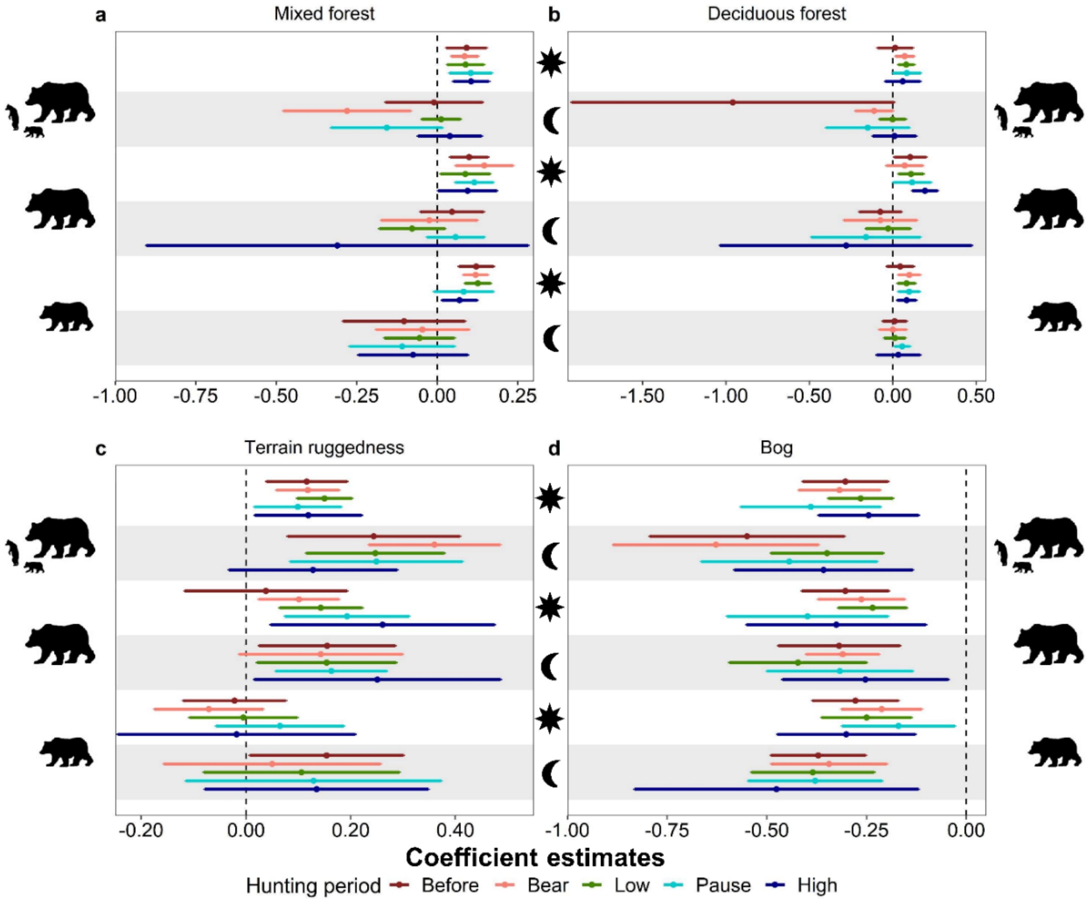

Figure S3. Coefficient estimates for a) the selection of mixed forest, b) the selection of deciduous forest, c) the selection of terrain ruggedness and d) the selection of bog with 95% confidence intervals. The coefficients were estimated from integrated step-selection functions for female brown bears with dependent offspring (*n* = 18 bear-years), solitary females (*n* = 17 bear-years) and subadult females (*n* = 18 bear-years) for day and night in south-central Sweden, 2016-2019. The coefficients were estimated for each hunting period: before hunting (red), bear hunt (pink), low intensity moose hunt (green), pause (cyan) and high intensity moose hunt (dark blue). Other parameters are presented in Fig. 3 (main document), Appendix S1: Tables S6 and Appendix S1: Figure S4.

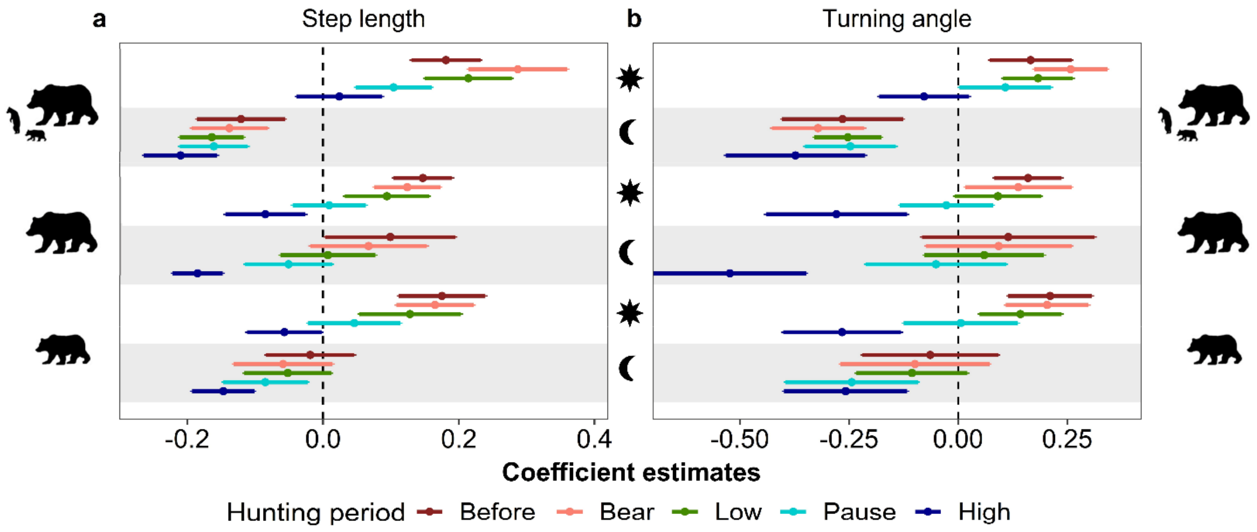

Figure S4. Coefficient estimates for a) the log step length and b) the cosine of turning angle with 95% confidence intervals. The coefficients were estimated from integrated step-selection functions for female brown bears with dependent offspring (*n* = 18 bear-years), solitary females (*n* = 17 bear-years) and subadult females (*n* = 18 bear-years) for day and night in south-central Sweden, 2016-2019. The coefficients were estimated for each hunting period: before hunting (red), bear hunt (pink), low intensity moose hunt (green), pause (cyan) and high intensity moose hunt (dark blue). Other parameters are presented in Fig. 3 (main document).

Table S6. Coefficients of movement parameters with 95% confidence intervals estimated from integrated step-selection functions for female brown bears with dependent offspring (*n* = 18 bear-years), solitary females (*n* = 17 bear-years) and subadult females (*n* = 18 bear-years) for day and night in south-central Sweden, 2016-2019. The coefficients were estimated for each hunting period: before hunting (red), bear hunt (yellow), low intensity moose hunt (green), pause (blue) and high intensity moose hunt (purple). The coefficients presented in this table were estimated from the bear habitat iSSF models.

		Step length [log(m)]		Turning angle [cos(rad)]	
		Day	Night	Day	Night
Females with dependent offspring	Before	0.225 ± 0.054	-0.099 ± 0.066	0.181 ± 0.096	-0.275 ± 0.139
	Bear	0.338 ± 0.078	-0.107 ± 0.060	0.263 ± 0.078	-0.326 ± 0.111
	Low	0.279 ± 0.076	-0.144 ± 0.048	0.197 ± 0.082	-0.255 ± 0.078
	Pause	0.167 ± 0.066	-0.131 ± 0.049	0.123 ± 0.113	-0.243 ± 0.108
	High	0.065 ± 0.067	-0.177 ± 0.054	-0.068 ± 0.105	-0.374 ± 0.162
Solitary females	Before	0.199 ± 0.044	0.124 ± 0.097	0.158 ± 0.073	0.117 ± 0.197
	Bear	0.180 ± 0.054	0.094 ± 0.090	0.148 ± 0.125	0.091 ± 0.169
	Low	0.144 ± 0.069	0.047 ± 0.074	0.103 ± 0.099	0.057 ± 0.138
	Pause	0.060 ± 0.056	-0.019 ± 0.066	-0.018 ± 0.106	-0.042 ± 0.160
	High	-0.017 ± 0.048	-0.125 ± 0.039	-0.264 ± 0.161	-0.504 ± 0.166
Subadult females	Before	0.213 ± 0.067	-0.001 ± 0.065	0.216 ± 0.094	-0.061 ± 0.152
	Bear	0.205 ± 0.059	-0.035 ± 0.073	0.211 ± 0.094	-0.099 ± 0.168
	Low	0.191 ± 0.081	-0.029 ± 0.066	0.159 ± 0.098	-0.113 ± 0.132
	Pause	0.094 ± 0.071	-0.059 ± 0.064	0.018 ± 0.134	-0.239 ± 0.154
	High	-0.013 ± 0.061	-0.118 ± 0.045	-0.262 ± 0.137	-0.254 ± 0.138

Section 5: Post-hoc movement iSSF

Following our habitat selection analyses, we decided to investigate the brown bears response to moose hunting. iSSF can also be used to conjointly investigate movement and resource selection (Avgar et al. 2016); however, we could not investigate habitat specific movement in our original iSSF and included the movement parameters as controls (Signer et al. 2019, Fieberg et al. 2021), which is essential because bears modify their activity patterns during the fall. Investigating movement in an iSSF framework requires extracting resources at the start of each step and adding interactions with movement parameters and habitat variables, whereas resource selection requires the extraction of resources at the end of each step (Signer et al. 2019, Fieberg et al. 2021).

In this article, it was not possible to build a model with both habitat-specific movement and resource selection because we split our analyses according to time of day and it becomes an issue for the steps the overlap the onset of legal hunting hours. Thus, the start and end of these steps were recorded in different time periods and including habitat specific movement and resource selection in the same model could have led to bias estimates. We could also not create separate models for each demographic groups and pooled all individuals due to convergence issues; however, this is not an issue because the movement response to a disturbance is similar in all demographic groups (Ordiz et al. 2013). Therefore, we estimated the movement response to the probability of moose kill at the population levels with separate models for day and night during each of the hunting period with the structure described in Table 1 in the main document.

We fitted tentative Gamma and Von mises distributions for step length and turning angle for moving steps only (moving step = hourly displacement of > 40m) with all individuals combined with the *fit_distr* function [*amt* package; (Signer et al. 2019)]. We used the coefficients for the log of step length as modifiers of the original Gamma shape, whereas the coefficients for the cos of turning angle were used as modifiers of the original Von mises concentration parameters (Fieberg et al. 2021).

$$\text{Shape(RSFhunt)} = S_0 + \beta(\text{log step length}) + \beta(\text{log step length:RSFhunt}) \cdot \text{RSFhunt} + \beta(\text{log step length:RSFhunt}^2) \cdot \text{RSFhunt}^2$$

Where S_0 is the original shape parameter, β(log step length), β(log step length:RSFhunt) and β(log step length:RSFhunt^2) are the model coefficient for the log of step length and the interactions between the log of step length and the probability of moose kill (RSFhunt) and its quadratic term (RSFhunt^2).

$$\text{Kappa(RSFhunt)} = K_0 + \beta(\text{cos turning angle}) + \beta(\text{cos turning angle:RSFhunt}) \cdot \text{RSFhunt} + \beta(\text{cos turning angle:RSFhunt}^2) \cdot \text{RSFhunt}^2$$

Where K_0 is the original concentration parameter (i.e., kappa), β(cos turning angle), β(cos turning angle:RSFhunt) and β(cos turning angle:RSFhunt^2) are the model coefficient for the cos turning

angle and the interactions between the cos turning angle and the probability of moose kill (RSFhunt) and its quadratic term ($RSFhunt^2$).

We calculated the expected speed (m/h) of female brown bears when traveling through low and high probability of moose kill (10% and 90% quantiles) by multiplying the scale and shape of updated gamma distributions for day and night during each hunting period (Appendix S1: Table S7). The updated Von mises distributions were generated using the *dvonmises* function from the *circular* package (Agostinelli and Lund 2017). We did not need to generate updated Gamma distributions of step length, since we could obtain the mean expected hourly displacement by multiplying the updated shapes with the original scales.

Table S7. Mean expected hourly displacement (m/h) with 95% confidence intervals for female brown bears (n = 53) when traveling through areas with low and high relative probability of moose kills (RSFhunt) during day and night of each hunting periods in south-central Sweden during 2016-2019.

		**Low RSFhunt (10 % quantile)**			**High RSFhunt (90 % quantile)**		
		Expected hourly displacement (m/h)					
		Mean	Lower	Upper	Mean	Lower	Upper
Before	Day	523	513	534	626	585	667
	Night	450	434	467	473	433	513
Bear hunt	Day	545	530	561	614	573	654
	Night	435	417	453	460	422	497
Low intensity moose hunt	Day	528	516	540	580	547	613
	Night	436	423	449	447	419	474
Moose hunt pause	Day	493	483	502	551	510	593
	Night	412	400	424	444	416	572
High intensity moose hunt	Day	462	450	475	501	462	541
	Night	382	374	390	422	392	452

We similarly generated probability densities of turning angles when traveling through low and high probability of moose kill (10% and 90% quantiles) by using the updated Von mises distributions for day and night during each hunting periods (Appendix S1: Figure S5).

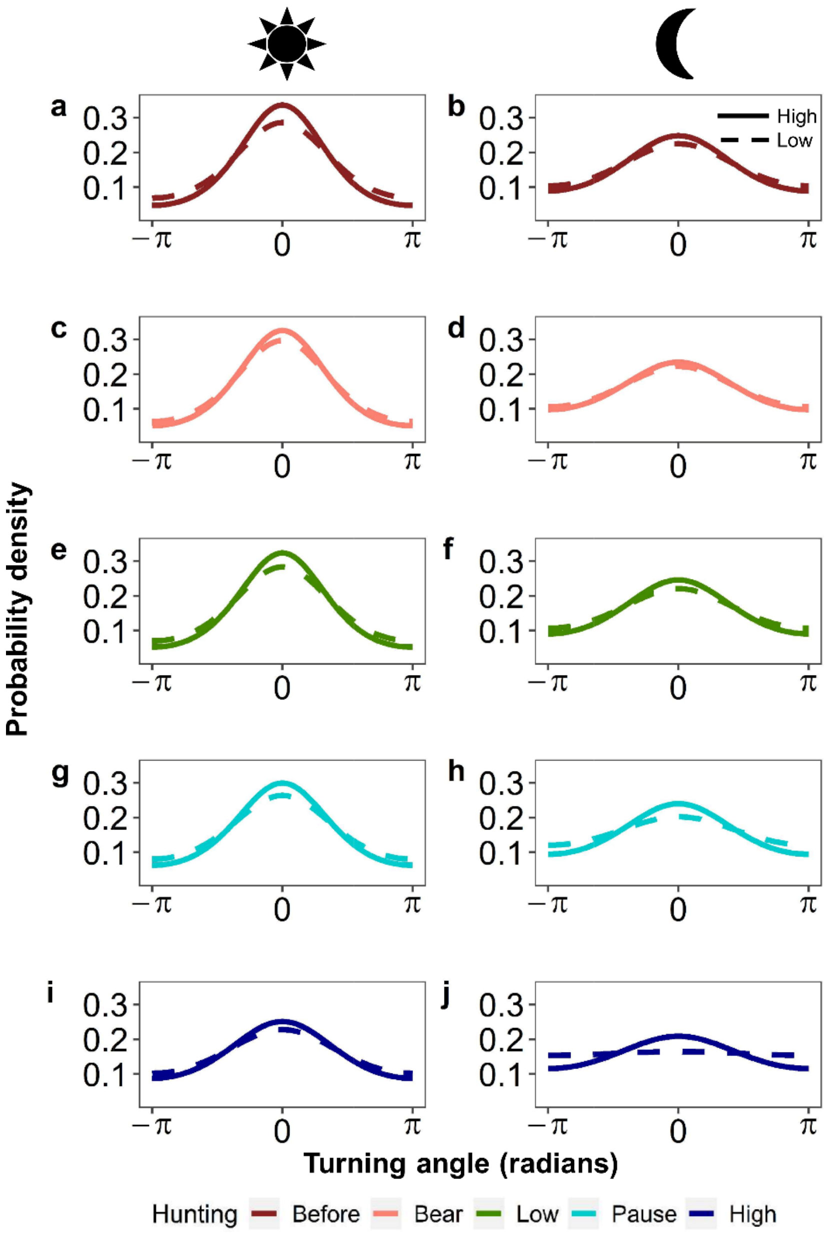

Figure S5. Probability densities of turning angles (radians) for female brown bears (n = 53) when traveling through areas with low (10% quantile) and high (90% quantile) relative

probability of moose kills (RSFhunt) during day and night of each hunting periods in south-central Sweden during 2016-2019. Before hunting = red, Bear hunt = pink, Low intensity moose hunt = green, Moose hunt pause = cyan and High intensity moose hunt = dark blue.

Brown bears moved faster and more directionally when traveling through areas with high probabilities of moose kill during day and night in most hunting periods, which supports our interpretation that moose hunters were perceived as a threat.

References

Agostinelli, C., and U. Lund. 2017. R package *circular*: Circular Statistics (version 0.4-93).

Avgar, T., J. R. Potts, M. A. Lewis, and M. S. Boyce. 2016. Integrated step selection analysis: Bridging the gap between resource selection and animal movement. Methods in Ecology and Evolution 7:619–630.

Burnham, K., and D. Anderson. 2002. Model selection and multi-model inference. Second. Springer-Verlag, New-York.

Fieberg, J., J. Signer, B. Smith, and T. Avgar. 2021. A ‘How to’ guide for interpreting parameters in habitat-selection analyses. Journal of Animal Ecology 90:1027–1043.

Mazerolle, M. J. 2020. Model selection and multimodel inference using the AICcmodavg package.

Ordiz, A., O.-G. Støen, S. Saebø, V. Sahlén, B. E. Pedersen, J. Kindberg, and J. E. Swenson.

2013. Lasting behavioural responses of brown bears to experimental encounters with humans. Journal of Applied Ecology 50:306–314.

Pitman, R. T., J. Fattebert, S. T. Williams, K. S. Williams, R. A. Hill, L. T. B. Hunter, H. Robinson, J. Power, L. Swanepoel, R. Slotow, and G. A. Balme. 2017. Cats, connectivity and conservation: incorporating data sets and integrating scales for wildlife management. Journal of Applied Ecology 54:1687–1698.

R Core Team. 2021. R: A Language and Environment for Statistical Computing. Vienna, Austria.

Signer, J., J. Fieberg, and T. Avgar. 2019. Animal movement tools (*amt*): R package for managing tracking data and conducting habitat selection analyses. Ecology and Evolution 9:880–890.
